# Retraction: Ñíguez Sevilla, B., et al. Nurse’s A-Phase–Silicocarnotite Ceramic–Bone Tissue Interaction in a Rabbit Tibia Defect Model. *J. Clin. Med.* 2019, *8*, 1714

**DOI:** 10.3390/jcm9061888

**Published:** 2020-06-17

**Authors:** JCM Editorial Office

**Affiliations:** MDPI, St. Alban-Anlage 66, 4052 Basel, Switzerland; jcm@mdpi.com

In the published paper [1], Figure 5 (five month) is similar to Figure 5 (six month) of the paper published in *Materials* [2]. After thorough investigation, the journal has therefore made the decision to retract the paper [1]. The authors were unable to provide an adequate explanation for the image discrepancies, resulting in unreliable data. The Editor-in-Chief and Academic Editor of the *Journal of Clinical Medicine* have checked the case, and the Editor-in-Chief has approved the retraction.

We apologize to readers of *Journal of Clinical Medicine*. MDPI is a member of the Committee on Publication Ethics and takes the responsibility to uphold strict ethical policies and standards very seriously.
